# Infiltrated Pits: Using Regional Groundwater Data to Estimate Methane Emissions from Pit Latrines

**DOI:** 10.3390/hydrology10050114

**Published:** 2023-05-19

**Authors:** Olivia Reddy, Mostaquimur Rahman, Anisha Nijhawan, Maria Pregnolato, Guy Howard

**Affiliations:** Department of Civil Engineering and Cabot Institute for the Environment, University of Bristol, Bristol BS8 1TR, UK

**Keywords:** groundwater, onsite sanitation, greenhouse gas emissions, pit latrine

## Abstract

On-site sanitation systems (OSS), such as pit latrines, are an important source of methane (CH_4_), with emissions increasing when they are wet, and this occurs when anaerobic conditions dominate. This paper presents the development of a model, which uses seasonal changes in groundwater to account for the fluctuating inundation of pit latrines, and, therefore, the associated CH_4_ emissions from varying degrees of anerobic conditions are examined. Given that observed timeseries of groundwater table depth at high enough spatial and temporal resolutions are often difficult to obtain in low- and middle-income countries (LMICs), inverse distance weighted (IDW) interpolation is used to generate values for a whole region, which is then used, alongside average pit latrine depth, to determine areas of pit latrine inundation. Outcomes are further informed with open-source contextual data, covering population, urban/rural split, and sanitation facility data, before using methodologies from the Intergovernmental Panel on Climate Change (IPCC) to generate CH_4_ emissions data. As a case study, we use data from Senegal to illustrate how this model works. Results show total CH_4_ emissions for the month of January to be ~1.69 kt CH_4_. We have also discussed the potential use of satellite remote sensing data in regions where access to historical groundwater data is limited. Understanding when the pit conditions are most likely to change could lead to incentives for better management strategies, as well as a reduction in CH_4_ production.

## 1. Introduction

On-site sanitation systems (OSS) are an important source of greenhouse gases (GHG), particularly CH_4_, which are driving global heating [1–3]. Referring to pit latrines, septic tanks, and other non-sewered sanitation, OSS is an integral part of faecal management, serving approximately 3.3 billion people [4] worldwide, the majority of whom live in low- and middle-income countries (LMICs). The interaction between pit latrines and groundwater has been widely explored in the realm of public health, mainly by assessing the effects that the percolation of leachate from pit latrines has on the quality of groundwater [5]. The risk of groundwater contamination is greater when pits are flooded [5], and this flooding also leads to CH_4_ production [3].

To determine the impact that OSS has on global emissions, it is essential to assess the quantity of GHG emissions they produce. The small number of studies that have been undertaken to capture this phenomenon tend to focus on GHG from septic tanks, and they do not consider the use of pit latrines [6]. This is despite the fact that pit latrines are used by roughly the same number of people globally as septic tanks, around 1.6 billion people [1,4]. The focus of septic tanks is largely a consequence of previous studies being undertaken in countries, such as the USA and China, where septic tanks are the more common choice of OSS [6].

There have been some studies where estimates for total CH_4_ from pit latrines have been presented. Reid et al. (2014) reported that 1% of total global CH_4_ emissions could be attributed to pit latrines alone [3]. van Eekert et al. (2019) found similar results, and when CH_4_ is converted to global warming potential, pit latrines were estimated to contribute to 0.3% of total global GHG emissions for 2014 [7]. Although measuring from EcoSan systems, direct measurements made by Ryals et al. (2019) show that CH_4_ emissions from moister pile conditions produced significantly higher emissions than those with dry conditions [8]. Providing that the system provides anaerobic conditions, methanogenesis can take a few hours to days to occur [9].

These studies used different methods, with van Eekert and colleagues undertaking soil sample collection and comparing in vitro and in situ results, whereas Reid and colleagues used spatial modelling [3,7]. In contrast, Johnson et al. (2022) took a whole system analysis approach in a study of Kampala, Uganda, where rich data on sanitation type, usage, and systems were available [10]. They concluded that sanitation, in total, contributed around half of the total GHG emissions for the city, and, although they did not identify specific contributions from pit latrines, they noted that latrines that were inundated with surface or ground water had higher emissions.

In anaerobic conditions, more CH_4_ is produced, causing a greater warming potential than that produced in aerobic conditions. Increasing the water content of a system leads to a change in nutrient diffusion rates, and it can affect the metabolism of bacteria, increasing production of CH_4_ in anaerobic systems [7]. To address this issue, the Intergovernmental Panel on Climate Change (IPCC) offered different emission factors for wet pit latrines and dry pit latrines [2]. For pit latrines, the methane correction factors (MCF) can range from 0.05 to 1.0, as defined by the IPCC [2], depending on the depth of the latrine relative to the groundwater table, and, therefore, this is also related to the associated moisture content of a system. The MCF is used to determine how much of a system is subject to anerobic digestion, with those systems, which are deemed wetter, producing more CH_4_ overall.

Generating data from empirical studies is essential and should be continued. However, because it is time consuming, expensive, and impractical to rely on extensive direct measurements from pit latrines and recorded inundation in most cases, other methods of deriving location specific, seasonal emissions measurements are required. Since pit latrines can be infiltrated by groundwater, it can be assumed that the seasonal depth of the groundwater in an area can be used to determine when the pit will be wet or dry. The groundwater data that underpin the study by Reid et al. (2014), although using a total of 1,603,781 groundwater collection sites, had a scarcity of data points across LMIC, especially across Africa, with only 431 sites [3,11]. This low amount of data is often due to a lack of funding and resources to undertake this monitoring [12,13].

Given the need to better understand inundation, in this paper, we develop a model to estimate likely inundation of pit latrines by groundwater, based in the assumption that groundwater level can be used to determine the likelihood of inundation, and, therefore, it can influence what type of emission factors, as defined by the IPCC, are used. We believe such an approach offers a more nuanced view of seasonal fluctuations of pit latrine status—whether wet or dry—across a region.

There are two possible ways in which this data can be used to assess sanitation. The main aim of this model is to illustrate how they can be used to generate predicted CH_4_ emissions data from pit latrines with greater certainty than has been previously achieved. Secondly, they can be used as tools to inform management strategies, such as the best time to empty pit latrines to avoid groundwater inundation, by understanding when this is most likely to happen and in which areas. Throughout the paper, data from across Senegal are used to illustrate how the model works.

## 2. Materials and Methods

**Figure 1.**
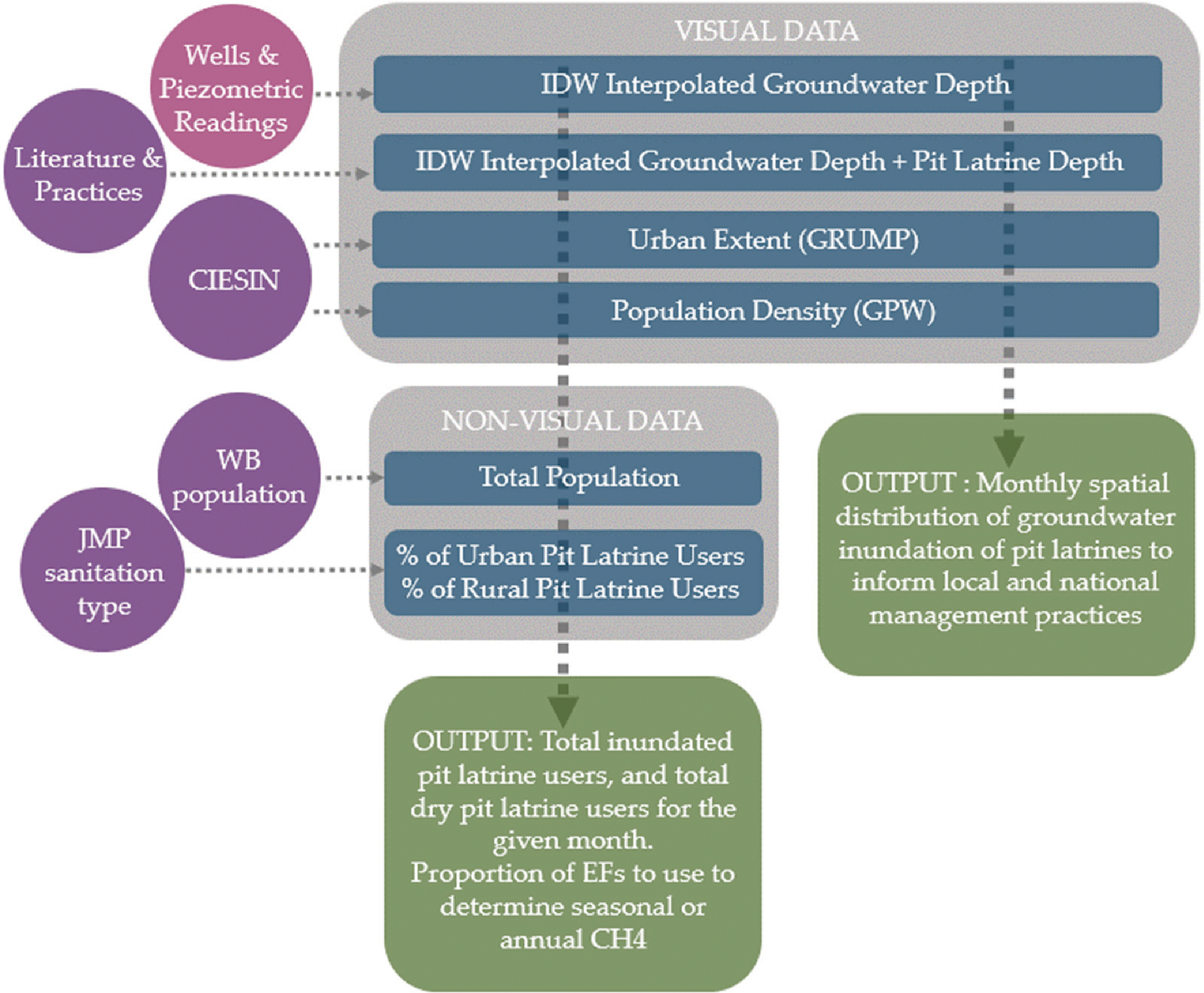
Visualization of the model’s architecture, including input data, sources, and outputs.

The data required to determine the seasonal extent of groundwater infiltration of pit latrines can be divided into two main categories: (1) seasonal groundwater data and (2) contextual data (demographic, latrine location, and emissions factors). For (1), as the time of year for different seasons, and the type of season varies across the world, seasonality is broken down into monthly segments. The use of IPCC emission factors (EF) to determine overall CH_4_ associated with groundwater inundation are also described [2]. A visualization of the model and its components can be seen in Figure 1.

The model is based on Geographical Information Systems (GIS). The Quantum Geographic Information System (QGIS^™^) [14] was selected because it is open access, allowing all users to download it freely.

### 2.1. Seasonal Groundwater Data

To gain an understanding of the seasonal variation of groundwater level, historical groundwater data are gathered from government departments, universities, or other research groups, which hold them on record. These data are then gathered in a single database, converted to water table depth (m), and separated by month. Because these data show individual data points of either well dipping or piezometric readings, the data are subjected to Inverse Distance-Weighted (IDW) interpolation to generate values across the whole country for each month. IDW interpolation and Kriging methods were considered for analysis because they are similar techniques, both using mathematical functions, with the latter using geostatistical methods [15]. Studies show similar results using both techniques when verified with additional empirical data collection [16,17]. In this study, IDW interpolation was used because this function is embedded within the chosen QGIS software.

In this case, the historical groundwater data come from a large data set, consisting of 1697 readings of the static level of wells across Senegal between the years 1939 to 2006, accessed by Direction de la Gestion et de la Planification des Ressources en Eau (DGPRE; Directorate of Water Resources Management and Planning), on 1 January 2022.

### 2.2. Pit Latrine Inundation

The depth of a pit latrine can vary from location to location. However, recommendations from the literature state that a pit latrine should be at least 3 m deep, and it should preferably be deeper [18]. Therefore, this study uses 3 m as the average pit latrine depth. This choice is in line with the literature and the emissions study by Reid et al. (2014), where a depth of 2.5 ± 0.5 m was used [3]. Pit latrine inundation was determined by generating a new layer on QGIS to show which pixels of the IDW interpolation were shown as less than 3 m in depth. The pixels, which are shown to be less than 3 m in depth, are then coloured to “red” to show the areas of pit latrine inundation for that month.

### 2.3. Contextual Data

To determine how many people are affected by pit latrine inundation, widely available population data were used. This contextual data gathered include total population, percentage of pit latrine users, and urban/rural split. We assume, in our model, that these data do not change seasonally, although we recognise them as dynamic. It is noted that, for this paper, additional information used to define a pit latrine, such a lined/unlined, is not used. Here, the data used are from the World Bank and Joint Monitoring Programme, where they are simply designated as “pit latrines”.

**Total urban and rural population** are calculated from population and urban/rural spilt data from the World Bank, with the most recent updates for both made in 2021 [19]. An example of this data can be seen in Table 1.

#### Percentage cover of pit latrine use.

Information for sanitation type, and, therefore, pit latrine use, is extracted from the Joint Monitoring Programme [20]. The data are split into rural and urban categories, and they are divided by facility type into latrine, septic tank, and sewer categories. An example of the values for latrines can be seen in Table 1.

**Urban/rural distribution** is essential, as the number of pit latrine users in each population type is different. To determine the urban/rural spilt in an area, the *“Global Rural-Urban Mapping Project (GRUMP), version 1”* map from The Center for International Earth Science Information Network (CIESIN) [21,22] was used. This shows the urban extent of settlements based on buffered settlement points and the presence of night lights.

**Population density** is integral for determining the number of latrine users in a given area. Based on population registers and national censuses, the *“Gridded Population of the World (GPW), version 4”*, freely available from CIESIN [23], was used. As a heatmap, these data generate values across the world for the number of persons per square kilometer.

The four data sets described above were used together to generate results, using the IDW interpolation as a gridded mapped area, where each pixel is considered separately. Firstly, the population within a pixel is determined by:

(1) $$PX_{n}=Pd\times B$$

where PX is the total population of the pixel, n is the pixel number, Pd is the population density in the pixel, and B is the total count of 1 km^2^ boxes, represented by that population density. Where more than one population density value is present in a single IDW pixel, the total km^2^ represented by each density value is taken into consideration, and the sum of each Pd value is multiplied by its corresponding count of 1 km^2^ boxes, represented by that population density. This gives the total population of the pixel.

To then generate the total number of pit larine users, Equation (2) is applied:

(2) $$\left(P_{U}\times PL_{U}\right)+\left(P_{R}\times PL_{R}\right)=PL_{T}$$

$P_{U}$ and $P_{R}$ are the urban and rural populations of the pixel, respectively, where $PL_{U}$ and $PL_{R}$ are the percentage cover of pit latrine users in urban and rural areas as defined by the Joint Monitoring Programme (JMP), and $PL_{T}$ is the total number of pit latrine users in the pixel. If multiple population densities are represented, then this equation is expanded to include all densities present and the corresponding population of pit latrine users.

### 2.4. Emission Factors and Total CH_4_

In terms of total emissions, calculations are made by using the emission factors (EF) from the IPCC [2], generated by Equation (3):

(3) $${EF}_{j}=B_{0}\times {MCF}_{j}$$

$EF_{j}$ is the EF (CH_4_/kg BOD), j is the treatment/discharge pathway or system, $B_{0}$ is the maximum CH_4_ producing capacity (CH_4_/kg BOD), and $MCF_{j}$ is the methane correction factor (MCF) fraction.

Default $B_{0}$ for domestic water is given as 0.6 kg CH_4_/kg BOD and 0.25 kg CH_4_/kg COD, respectively, as “based on expert judgement by lead authors” in Chapter 6 of the IPCC [2].

When referring to j, this paper considers the values given to latrine systems. As shown in Table 2, the IPCC breaks down types of latrines into three distinct categories, with groundwater features described as either higher or lower than the latrine [2].

The emission factors were then entered into Equation (4), based on the IPCC methodology [1,2], adjusted for monthly values, rather than annual, to determine the estimated CH_4_ emissions from pit latrines of a given area for a defined month:

(4) $${CH}_{4}=P\times BOD\times 0.001\times d\times EF$$

CH_4_ is the total methane emissions from that given area (kg CH_4_/year), P is the population using the system in the given area, BOD is the country-specific biological oxygen demand (BOD) from excreta of each person in inventory year (g/cap/day), as defined by the IPCC [2], 0.001 is the conversion factor from grams BOD to kg BOD, d is the number of days in the given month, and EF is the emissions factor derived from the model described in results (kg CH_4_/kg BOD).

#### Total monthly CH_4_.

To generate the total CH_4_ from a given month, the total population of pit latrine users from red and blue pixels of the IDW interpolation are determined. The pixels are either considered *“inundated pit latrines”* or *“dry pit latrines”*. The associated EFs are then derived from the IPCC latrine descriptions in Table 2, where “inundated latrines” use type c factors, and “dry latrines” use type a or b factors, depending on the average household size of the country. Total annual CH_4_ is then determined by adding the monthly CH_4_ values together.

## 3. Results

The results from this study show how a novel model design (illustrated in Section 2), which uses spatial distribution, freely available contextual data, and available seasonal groundwater data, can generate information on regional pit latrine inundation.

**Figure 2.**
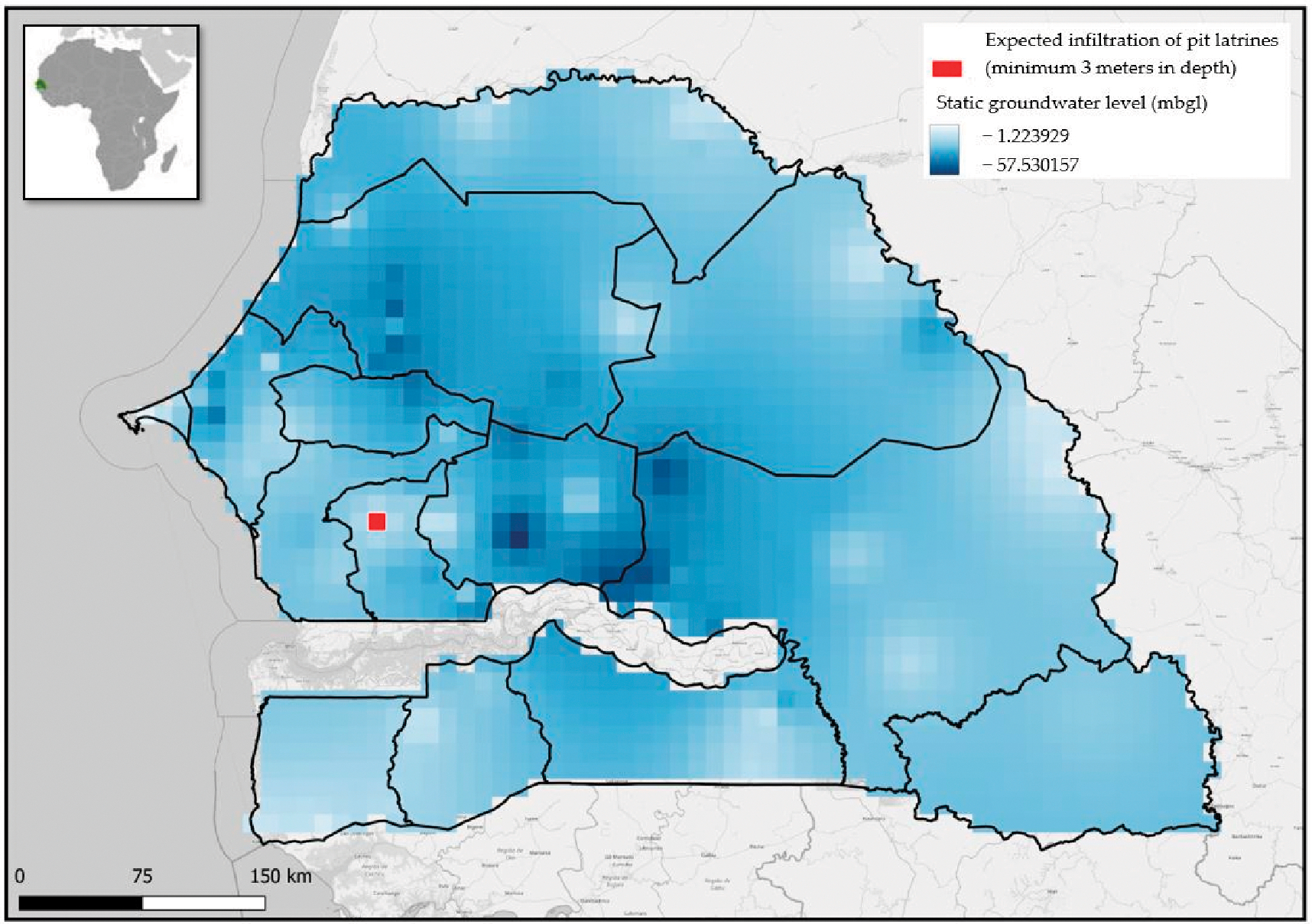
Output map for the country of Senegal in January using historical groundwater data from 1939–2006 and IDW interpolation. The red box shows where the infiltration of pit latrines of up to 3 m deep could be expected (meters below ground level—mbgl).

To illustrate the model application, results are derived from current information available to this research team from Senegal. This paper focuses on illustrating how the model works, whereas presenting country results is out of its scope; therefore, only data for the month of January are presented as an example. These data consist of 121 groundwater depth readings across Senegal, from 1939 to 2006 (from DGPRE). As the groundwater input data covers a large date range, the output for “January”, in this case, can be considered as an ~80-year average. Figure 2 shows the IDW interpolation map generated from this data, with the red square representing the location that is predicted to incur pit latrine inundation.

This map is considered the first output of this model, as it clearly illustrates geographically, across the region of Senegal, where pit latrine inundation by groundwater is likely to occur in January.

The second output showing predicted monthly CH_4_ emissions requires further calculations. In this case, the IDW inundation pixel size is approximately 11 km^2^. When calculated using Equations (1) and (2), this gives a total of 10,121.1 *“inundated pit latrines”* (red) users and 4,909,442.8 *“dry pit latrines”* (blue) users for the month of January (including both rural and urban users).

As the average household size in Senegal is ~8.7 persons [24], type (b) EFs are used for the dry proportion of the population. EFs for type (c) are used for the inundated latrine proportion of the population. The values that were entered into Equation (4) to determine the January CH_4_ emissions from pit latrines can be seen in Table 3. For *BOD*, 37 g/person/day is used, based on the literature presented by the IPCC for Africa. For d, as January has 31 days, 31 is used.

The total CH_4_ emissions from pit latrines in January are calculated to be ~1.69 kt CH_4_. Using this estimate from our model as an indication of monthly emissions and a global warming potential of 28 [25], total annual CH_4_ from pit latrines is estimated to be ~568 Kt CO_2_ e/year. A national audit for total sanitation emissions from Senegal [26] shows the country as producing 1723 Kt CO_2_ e/year, and on-site sanitation accounts for 1174 Kt CO_2_ e/year. Using the data on sanitation for Senegal for 2020 [20], *“improved latrine and other”* makes up 45% of total on-site sanitation. Applying this proportion to the total estimated on-site sanitation emissions indicates that pit latrines account for 528 Kt CO_2_ e/year. This is only 8% lower than the estimate calculated from our model, suggesting that, although these are crude calculations and there are more factors to consider, our model produces reasonable estimates.

It is important to note that this is a first estimate of the total CH_4_ emissions of Senegal, in January, using this model design. More work is required to verify the performance of this model and to quantify the uncertainties associated with these results.

## 4. Discussion

Our model provides a new tool to estimate likely changes in CH_4_ emissions in relation to changes in water content in pits, based on a groundwater model. By using local data and interpolating values between adjacent points to create a groundwater map estimate on potential, inundation can be created, providing new tools to support decision-making concerning issues, such as emptying pit latrines.

### 4.1. Benefits of This Model

The output maps can be used for two main purposes. Firstly, to determine the CH_4_ emissions for a region by using multiple EFs at varying degrees across the year, as well as to take into account seasonal variation. Secondly, they can be used to roughly forecast when pit latrines in certain areas are more likely to become inundated with groundwater, and, therefore, they can be used to develop management and emptying practices, which reflect this. Not only could this model be used as a means to manage GHG emissions, but it can also improve public health by ensuring pit latrines do not reach a stage where they overflow. Emptying latrines is a complex issue. Therefore, other associated issues would have to be considered to determine the capacity of a certain location to change its emptying practices. This includes, but is not limited to costs, safety of emptying and suitability of disposal/onwards treatment. Emptying must be considered in a holistic way, and this model aims to aid in a small part of this. Overall, this model provides a good generalisation of a region with the data that are available.

IDW interpolation is chosen because it allows a small accurate dataset to be interpolated across the desired area. By using this type of spatial interpolation, regions which have few data points are still able to achieve usable results. Ideally, the measurements should be taken at regular intervals across the desired region. However, as with any spatially driven work, the level of uncertainty within the results is dependent on the quality (and quantity) of the input data.

Although rainfall can cause ponding in latrines at times, this phenomenon occurs when there is no superstructure present. In this study, seasonal inundation of pit latrines due to groundwater is explored because it lends a more measurable and direct way to measure water inundation of improved pit latrines. This inundation comes from the rising of groundwater, often after the rainy season, assuming that the aquifer is isotropic.

Currently, whole-country GHG emissions estimates are more often than not based on emissions factors by the IPCC [2], where estimates are made for the percentage of the country that would be experiencing groundwater inundation across the year. This is shown in the way that the IPCC categorizes latrine types—it is performed by shallow or deep groundwater tables [2]. Providing figures that most accurately represent annual emissions should take into consideration seasonal change, which is not currently explored at present. Our model allows an annual emissions figure to be presented with greater certainty, backed-up with country-specific data.

Although there are discussions and disagreements within the literature around whether the EFs from the IPCC overestimate [27–29] or underestimate [10] the associated GHG emissions, these are used for this study because, overall, more research needs to be undertaken to determine otherwise. If these factors do change in the future, they can easily be changed within the model too, with the other factors remaining the same. This model is flexible and is designed to use the most recent data available. Likewise, there are differences in opinion on the dominance of anerobic digestion pathways within pit latrines [7,30]. However, as previously mentioned, more empirical data are required before any sure decisions are made.

While pit latrines are the focus of this paper, it is possible to assume that many septic tanks will not be perfectly built and managed, and, therefore, many of these will also be at risk of infiltration by groundwater [1,4]. For the case of Senegal, pit latrines only account for 29.3% of the population’s sanitation usage [19,20]. In a country where septic tanks are more widely used (36.7% in the case of Senegal [19,20]), it would be valuable to understand the percentage of these which are both working and managed as designed, as well as the percentage of those that are not and, therefore, what impact this may have on total CH_4_ emissions associated with onsite sanitation. It is possible that most septic tanks may be contained well enough so that the contents are always submerged due to flush water, and, therefore, this mapping of groundwater would not be required with regards to emissions.

Other studies have put forward the hypothesis that, if a reduction in GHG emissions can be shown, then sanitation projects may be able to access climate financing [31]. OSS management strategies could be informed by this model as a way of reducing emissions and be part of a programme of action able to access climate financing by demonstrating effective CH_4_ reduction strategies. Additionally, pathways to accessing climate financing could open areas that incorporate this if it can be shown to be reducing GHG emissions in the area.

### 4.2. Model Limitations

Other factors that are likely to have an impact on total production of CH_4_ include the anal cleansing method (whether water or paper is used), differences in practices/usage in urban and rural settings, the total sludge content in each pit latrine, and the level of sludge stabilisation. Although this may be the case, it is impossible to monitor each of these factors when working on this larger scale. This model uses an approach where data that are readily available are used to create *“best estimates”,* rather than undertaking new data collection onsite. However, we acknowledge the importance of these other factors and, therefore, encourage extra data collection where possible.

Although this model is grounded in real data, and it uses logical steps to generate results, there are still many uncertainties within it. Much of these uncertainties stem from the type of data which are available.

The latest update for urban extent data by CIESIN was received in 2000 [21,22]. In Senegal, this means that the map used is not only 23 years out of date, but it is based off a population size of 9,704,287 [19], rather than the current population of 16,876,720 [19]. The fraction of people living in urban areas has also increased, from approximately 45.5% to 49% today [19,21,22]. However, when a more recent population density map [32] from the 2021 census is overlayed, clusters form in the same areas. As this map shows population clusters, not urban extent, it was not used for the model, but to verify what was used. It is, therefore, possible to assume that much of the spatial distribution of the population has remained similar, even if it has increased in number. For future work, correction factors will be developed for each country, based off the most recent census data.

Although this data may not be as recent as desired, the fact that population distribution, urban extent, and seasonality are considered alongside groundwater, grounds the EFs, which are used much more so than current methodologies.

With regard to the hydrology of a given region, this model assumes that all aquifers are isotropic, and, therefore, groundwater level has a direct seasonal rise and fall, which will affect pit latrine inundation. However, this assumption cannot be extended globally, and, therefore, local knowledge of an area’s hydrology is important to determine how much of a driving force groundwater infiltration plays within that region. For example, in Uganda, Taylor and Howard (1999) [33] note that preferential pathways of subsurface flow commonly occur due to the influence of tectonic setting in the region. In this case, this model may not be appropriate because isotropic groundwater would not be the driving factor of inundation. Again, this model offers a good generalization of a region, without delving into specifics of an area. It is, therefore, suggested that, if this model is to be used to help inform management, local knowledge and understanding of hydrology should also be considered.

Perhaps the most limiting factor of this model is the use of regional historical groundwater data. For the considered case of Senegal, obtaining a data set of over 1500 data points for a single region is a remarkable achievement. In the study by Reid et al. (2014), the whole continent of Africa is represented by only ~431 data points [3,14]. Unfortunately, this dearth of data is the case for many LMICs, as access is often limited, or often behind a paywall, when the data have been collected. For this data to prove robust, there must be sufficient data across the area for each season, with several data points in each instance. This constraint is by far the most limiting factor of using this method of groundwater projection. Many uncertainties lie within the total number of data points and the size of the area they are interpolated over.

The success of this model is based on access to groundwater data from the study country. To overcome this, we have begun to investigate other ways to access non-traditional groundwater data, which do not require years of field work or monetary resources to generate.

### 4.3. Future Use of Satellite Technologies

**Figure 3.**
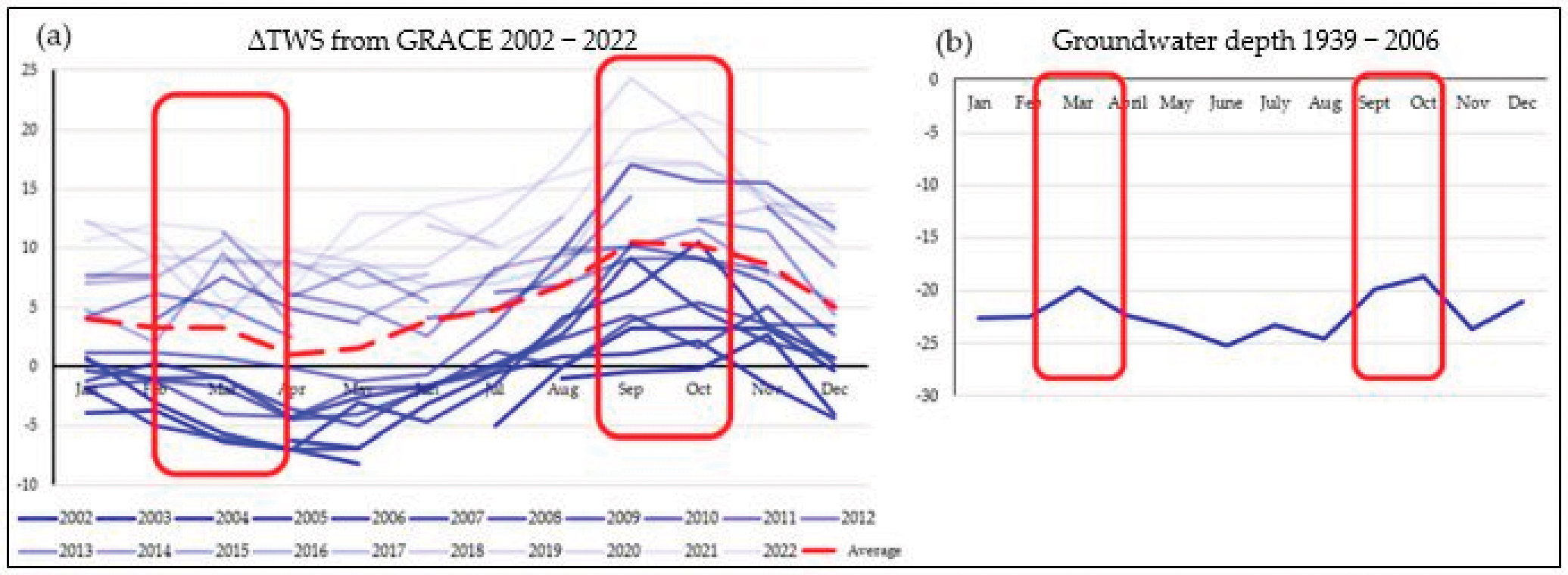
Mean groundwater depth was generated by using the data points within the area that the GRACE pixel covers. (**a**) Data from GRACE: note that GRACE data are represented as ΔTWS, not in groundwater depth. The average ΔTWS for each month from 200–2022 is presented as the red dotted line. (**b**) Measured average groundwater depth using the average from 1939–2006. For both (**a**,**b**), the seasonal trends where water in the region is highest are highlighted by the red boxes.

The Gravity Recovery and Climate Experiment (GRACE) satellite is used to show groundwater trends over a larger area. GRACE [34] and its successor, GRACE-Follow On (or GRACE-FO) [35], provide near continuous monthly data on terrestrial water storage anomaly (ΔTWS) across Earth. The twin satellites monitor the change in gravity mass, and, therefore, there is a change in water storage within a given pixel (with a maximum resolution of ~400 km [36]). Although less granular than using empirical groundwater data, GRACE has often been used to verify groundwater research in terms of determining if the predicted groundwater results follow the same trend as monitored by GRACE satellite data [15]. In the context of Senegal, these mirroring trends can be seen in Figure 3.

Although the March peak seen in (b) is not as prevalent in (a), the trend is still visible. As averages over large time periods are used, as well as data, which do not directly show groundwater level, this uncertainty is to be expected. At this stage, no correlation was undertaken due to the GRACE data not representing groundwater depth. However, the trend is clear enough to suggest that further work should be undertaken in this direction.

Data from GRACE have also been used to study trends in groundwater where there is no empirical data [37]. Many of these studies have used GRACE to provide useful descriptive information where any other data are lacking [15]. In terms of TWS, this is clearly demonstrated by the data from Senegal.

To generate useful data in terms of groundwater, TWS data from GRACE can be used in combination with the Global Land Data Assimilation System (GLDAS) [16,38,39]. GLDAS models a multitude of land surface variables, including surface water storage and soil moisture [40], and, therefore, Equation (5) applies:

(5) $$\Delta TWS=\Delta W_{sws}+\Delta W_{sm}+\Delta GWS$$

ΔTWS is the total water storage anomaly derived from GRACE, and $\Delta W_{sws},$ $\Delta W_{sm}$, and ΔGWS represent the total anomalies of surface water storage, soil moisture storage, and groundwater storage, respectively.

Therefore, to solely derive the change in groundwater storage ΔGWS from month-on-month, the following equation can be used:

(6) $$\Delta GWS=\Delta TWS-\Delta W_{sws}-\Delta W_{sm}$$

ΔGWS describes the month-on-month trends of change in groundwater, not the absolute value of groundwater storage.

GRACE, in combination with GLDAS, provides continuous month-on-month data, over the same region, for ΔGWS since 2002. The nature of this evenly continuous data means that GRACE can be especially useful for determining trends across the region, albeit at a larger scale. For example, the months where groundwater table depth is the highest may have changed across a 15-year period, or these seasons may be regular. Whereas, data from GRACE can always accomplish this, and in-site measurements are rarely undertaken over such a long period.

It is, therefore, possible to recommend that in situ and satellite measurements are used to support each other [41]. Over the large regions defined by GRACE, data can be used to determine what areas of a country are more likely to receive large fluxes in groundwater level, as well as in what seasons. Whereas, in situ data will be able to provide more local information on exactly what communities may be at risk of inundation compared to others. Where little to no historical groundwater data exist, this method of using GRACE allows data scarce areas to still gain from this groundwater infiltration knowledge, albeit at a lesser resolution.

Some studies have compared in situ groundwater measurements [38,42] or have used interpolation of groundwater data to verify the results that they have obtained from GRACE [16]. Similarly, a comparison assessing the discrepancies across the two methods for assessing groundwater data is required. Finding a region which is both rich in historical groundwater measurements that overlap the years that GRACE has been running and has a significant percentage of the population that utilize pit latrines may prove to be difficult.

### 4.4. Validation and Future Work

This model requires validation using empirical CH_4_ emission data collected from pit latrines to determine how similar direct measurements are to model predictions. Under an ongoing project funded by the Bill & Melinda Gates Foundation (grant No: INV-015713), steps are being undertaken to do just this, including assessing emptying practices and the amount of sludge in OSS in both urban and rural regions. The initial steps of this empirical data collection and research from Senegal were presented by Ngom et al. (2022) [43].

However, as the model stands, without validation, we believe that this offers a more accurate way than current methods to estimate CH_4_ emissions from sanitation, as seasonality is considered completely supported by regional data. As trends in wetness are determined by seasons, it is essential to take them into account. By using groundwater levels to determine this, we have chosen a data set that has a stable time trend, as well as a relatively simple hypothesis that assumes that, when groundwater level reaches within the depth of a standard pit latrine, it will cause the contents to generate anaerobic conditions. Other hydrological data, such as rainfall, were considered. However, it is difficult determine how, where, and at what frequency rainfall has a direct impact on the contents of a pit latrine.

## 5. Conclusions

A new model has been created and is at the late development stage, which allows regional and seasonal estimates of CH_4_ emissions from pit latrines to be made. The novel part of this study is that seasonal groundwater inundation of pit latrines is taken into consideration for the first time.

This study focuses on using emissions data based on the current IPCC methodologies and the given EF. This study shows that, due to the limited availability of empirical groundwater data, other methods/sources should be used to enhance results if possible. Satellite data, such as GRACE, may not be granular, but they can provide up-to-date, current data, to complement any empirical data to generate a “best guess”.

Studies have shown [13] that these results can be improved by using and collecting location based empirical data. By using the methods outlined in this paper in conjunction with specific empirical data, more accurate quantities of seasonal emissions can be modelled, which more closely consider local environmental conditions, and thus they improve the model granularity.

The model presented in this paper shows potential in being able to model likely pit inundation over large regions, but it requires further development and validation. This paper also discusses a novel way to explore the use of satellite data within the field of sanitation. This knowledge may then be used to influence future on-site sanitation management strategies in the future.

## Figures and Tables

**Table 1.** Contextual data for Senegal.

	Senegal

^1^ Population	16,876,720
^1^ Urban/Rural Split	51/49
Urban Population	8,269,592.8
Rural Population	8,607,127.2
^2^ [Rural] Latrine %	30.41
^2^ [Urban] Latrine %	28.13

1 World Bank, 2021 [15],

2 Sanitation Facility Type, % Cover [16].

**Table 2.** Default MCF values and resultant EF for domestic wastewater by type of treatment system and discharge pathway, adapted from Chapter 6 of the IPCC [2].

Latrine Description	MCF (Range)	EF (kg CH_4_/kg BOD)	EF (kg CH_4_/kg COD)
(a) Dry climate, ground water table lower than latrine, small family (three to five persons)	0.1 (0.05–0.15)	0.06	0.025
(b) Dry climate, ground water table lower than latrine, communal (many users)	0.5 (0.4–0.6)	0.3	0.125
(c) Wet climate/flush water use, ground water table higher than latrine	0.7 (0.7–1.0)	0.42	0.175

**Table 3.** Data used and results of total January CH_4_ emissions from pit latrines for Senegal.

	Population	EF (kg CH_4_/kg BOD)	CH_4_ Emissions (kg CH_4_/Month)

Inundated Pit Latrines	10,121.1	0.42	4876
Dry Pit Latrines	4,909,442.8	0.3	1,689,339

## Data Availability

No new data were created or analyzed in this study. Data sharing is not applicable to this article.
